# Synthesis of γ-Nitro Aliphatic Methyl Esters Via Michael Additions Promoted by Microwave Irradiation

**DOI:** 10.3390/molecules14041595

**Published:** 2009-04-21

**Authors:** Jaime Escalante, Francisco D. Díaz-Coutiño

**Affiliations:** Centro de Investigaciones Químicas, Universidad Autónoma del Estado de Morelos. Av. Universidad No. 1001, Col. Chamilpa, C.P. 62210 Cuernavaca, Mor., Mexico; Email: dadico@ciq.uaem.mx (F-D.D-C.)

**Keywords:** Microwave irradiation, α, β-Unsaturated, Michael additions, γ-Nitro aliphatic methyl esters, Catalysis

## Abstract

A simple and efficient protocol has been developed for the direct synthesis of γ-nitrobutyric acid methyl esters under microwave irradiation. This methodology reduces reaction times from days to minutes, compared to conventional conditions. Additionally, these conditions increased yields and provided cleaner reactions.

## 1. Introduction

Microwave (MW) irradiation is widely used to promote chemical reactions and a number of reviews have advocated the use of MW technology in organic synthesis [1,2]. There are more than 3,000 documented examples of Microwave Assisted Organic Synthesis (MAOS) reported by both academic and industrial laboratories, which suggests that most chemical transformations can be carried out successfully under microwave conditions. The result of this has been a diverse range of chemical transformations, successfully performed through microwave irradiation, including cycloaddition, elimination, substitution and addition reactions [3,4,5,6,7,8,9,10,11,12,13,14]. Microwave-assisted heating under controlled conditions is an invaluable technology because it often dramatically reduces reactions times, typically from days or hours to minutes or even seconds [15,16,17,18,19]. The acceleration of reactions by MW exposure results from material-wave interactions leading to thermal effects connected to the intervention of “hot spots” (localized microscopic high temperatures) and specific (non-thermal) effects [20]. In the case of the specific (non-thermal) MW effects it has been found that reactions proceed with considerable lower yields under similar thermal conditions demonstrating that the effect of MW is evidently not purely thermal. These specific non-thermal effects of MW are increased when the polarity of a system is enhanced. In the Michael addition the rate-determining step consist in the nucleophilic addition on carbon-carbon double bond of α,β-unsaturated carbonyl compound. One can expect important specific MW effects due to the enhancement of polarity of the system during the reaction progress, which is provided by ionic dissociation of the ions pairs from the ground state of the reaction towards the transition state, which is more polar due to the negative charge delocalization. The more important stabilization of the transition state by dipole-dipole electrostatic interactions with the electric field is therefore responsible for an enhancement of reactivity by a decrease of the activation energy [21,22].

The reaction of functionalized nitroalkanes with electrophiles such as Michael acceptors is one of the most exploited procedures for the formation of new carbon-carbon bonds [23,24,25,26,27]. The versatility of this reaction is due to the variety of Michael donors and Michael acceptors (α,β-unsaturated compounds) that can be employed. The fact that the nitro functionality can easily be transformed into an amine, an oxime, a ketone, or a carboxylic acid, provides a wide range of synthetically interesting compounds [28,29,30]. Additionally, the carbon-carbon bond formation can potentially be made in a diastereo- or enantioselective fashion through the use of modern techniques applicable to asymmetric synthesis, affording optically active derivates. 

As a part of one of our projects aimed at the development of biocatalysts for the resolution of racemic amino methyl esters [31], our research group has developed a procedure for the preparation of a series of nitro methyl esters (**3**, **5**, **8**, and **11**-**13**), derived from 1,4-addition of nitroalkanes (**2** and **10**) to methyl crotonate (**1**), methyl acrylate (**4**), and methyl methacrylate (**7**), and catalyzed by 1,8-diazabicyclo[5.4.0]undec-7-ene (DBU) [32] under microwave irradiation. The addition reactions were carried out in nitromethane or nitroethane in a monomode CEM Discover microwave apparatus, in a sealed system, in vessels rated for high pressure. In every case the experiments were performed on a 10 or 20 mmol scale. 

## 2. Results and Discussion

### 2.1. Preparation of methyl 3-methyl-4-nitrobutanoate *(**3**)*

**Scheme 1:**
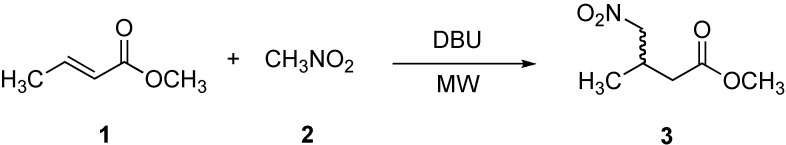
Preparation of methyl 3-methyl-4-nitrobutanoate (**3**).

In an initial study, the reaction between methyl crotonate (**1**), nitromethane (**2**), and DBU was carried out under the method described by Silverman (Scheme 1, Table 1, entry 1) [33], providing the γ-nitro aliphatic methyl ester **3** in 72 % yield after a prolonged period of time (4,320 min). It is worth mentioning that the product was also obtained under conventional conditions (70-75 ^o^C), albeit after a longer reaction time (Table 1, entry 2). Surprisingly, when the same reaction was performed under microwave irradiation, the reaction times decreased dramatically to 5 min, see Table 1 (entry 3) and gave better yields (≥ 98 %). To assess the number of equivalents of DBU required in this reaction, we carried out experiments using 0.1, 0.05, and 0.01 equivalents of DBU in 5 min (Table 1, entries 4, 5, and 6), with 0.1 equivalents being identified as the optimal concentration for this reaction. The low yield in entry 6 (0.01 eq of DBU) could be overcome by increasing the reaction time to 20 min (Table 1, entry 7, ≥ 97 % yield). In order to demonstrate the effect of microwaves on the 1,4-addition the reaction was carried out under conventional conditions (70-75 ^o^C) with 0.1 equivalents of DBU for 5 min (Table 1, entry 8). In this case, we found no product. Therefore, the observed rate-acceleration using microwaves demonstrating that the effect of MW is evidently not purely thermal.

**Table 1:** Effect of DBU on the 1,4–addition of nitromethane (**2**) to methyl crotonate (**1**).

Entry	Eq. DBU	Power (W)	Temperature (^o^C)	Time (min)	Yield 3 (%)
1	1.1	-	r. t.	4320	72
2	1.1	-	70-75	150	65 *^a^*
3	1.1	50	70-75	5	≥ 98 *^b^*
4	0.1	50	70-75	5	≥ 98 *^b^*
5	0.1	-	70-75	5	0 *^c^*
6	0.05	50	70-75	5	80 *^c^*
7	0.01	50	70-75	5	10 *^c^*
8	0.05	50	70-75	20	≥ 97 *^c^*

*^a^* Reaction was performed under conventional conditions and the yield was obtained after purification in column chromatography. *^b^* Reaction was performed under microwave irradiation and the yield was obtained after product purification by column chromatography. *^c^* Obtained by ^1^H-NMR.

### 2.2. Preparation of methyl 4-nitrobutanoate *(**5**)* and methyl 2-methyl-4-nitrobutanoate *(**8**)*

**Scheme 2:**
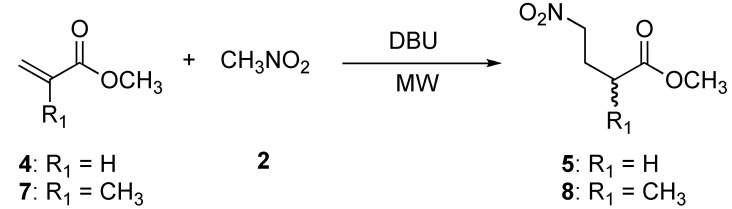
Preparation of methyl 4-nitrobutanoate (**5**) and methyl 2-methyl-4-nitrobutanoate (**8**).

We extended this protocol to the Michael addition of nitromethane (**2**) to methyl acrylate (**4**) and methyl methacrylate (**7**) (Scheme 2). 

**Figure 1:**
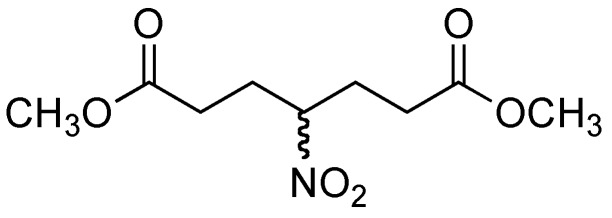
Double 1,4-addition product, compound **6**.

**Figure 2:**
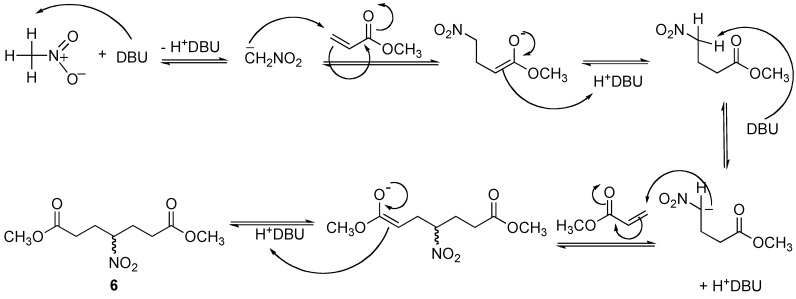
Proposed mechanism for the formation of the double 1,4-addition product **6**.

The addition of nitromethane (**2**) to methyl acrylate (**4**) provided compound **5** and one side product. TLC analysis of the reaction mixture after 30 min showed two products, the more polar product being the major component. After purification by column chromatography, the ^1^H- and ^13^C-NMR analysis indicated that the less polar product corresponded to a double 1,4-addition product **6** (Figure 1), whereas the more polar component was the desired product **5**, obtaned in 10 % and 69 % yield respectively. A proposed mechanism for the formation of dimeric product **6** is illustrated in Figure 2.

**Figure 3:**
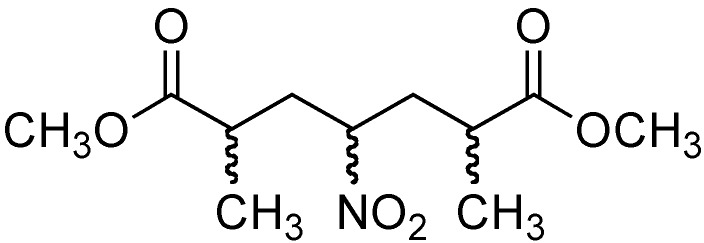
Double 1,4-addition product, compound **9**.

The addition of nitromethane (**2**) to methyl methacrylate (**7**) was carried out initially under conventional conditions in 4,320 min [33] (Table 2, entry 1). These conditions provided a mixture of products **8** and **9** (Figure 3) in 47 and 53 % yields, respectively. 

When the same reaction was performed under conventional conditions, the ^1^H-NMR analysis indicated a 27/73 ratio of the products **8**/**9** (Table 2, entry 2). In order to minimize the by-product 9 and to determine the effect of the microwaves in this process, the equivalents of DBU and the time were decreased to 0.1 eq and 5 min, respectively (Table 2, entry 3). Under these reaction conditions an **8**:**9**:**7** ratio of 45:45:10 was obtained. This result suggests that a thermal effect of the microwaves is essential in the formation of product **9**. In an effort to explore the specific (non-thermal) MW effect, we decided to change the base in this reaction, tetramethylguanidine (TMG, pKa~13.6) [34]. Interestingly, under MW irradiation the ratio of **8**:**9**:**7** was 72/28/0 (Table 2, entry 4). However, when the reaction was performed under conventional conditions, the ratio of **8**:**9**:**7** was 35/65/0 (Table 2, entry 5). This demonstrates that, in the Michael addition reaction, the formation of product **9** is due to a thermal effect, while the formation of product **8** can be explained by a specific (non-thermal) microwave effect [20,21].

**Table 2:** Formation of **8** and **9** by 1,4–addition of nitromethane (**2**) to Methyl Methacrylate (**7**).

Entry	Eq. Base	Power (W)	Temperature (^o^C)	Time (min)	8 / 9 / 7 (%) *^a^*
1	1.1 DBU	-	r. t.	4320	47 / 53 / 0
2	1.1 DBU	-	70-75	5	27/73/0
3	0.1 DBU	50	70-75	5	45/45/10
4	0.5 TMG	50	70-75	20	72 / 28 / 0
5	0.5 TMG	-	70-75	20	35/65/0

*^a^* Reaction was performed under microwave irradiation and the yield was obtained by ^1^H-NMR^.^

### 2.3. Preparation of methyl 3-methy-4-nitropentanoate *(**11**)*, methyl 4-nitropentanoate *(**12**)*, and methyl 2-methy-4-nitropentanoate *(**13**)*

**Scheme 3:**
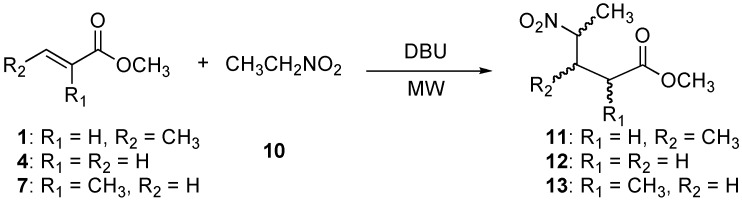
Preparation of methyl 3-methy-4-nitropentanoate (**11**), methyl 4-nitropentanoate (**12**), and methyl 2-methyl-4-nitropentanoate (**13**).

To expand the generality of this methodology, we also carried out the Michael addition of nitroethane (**10**) to the α,β-unsaturated esters **1**, **4**, and **7** under microwave heating (Scheme 3). 

First, the addition of nitroethane (**10**) to methyl crotonate (**1**) was performed under microwave irradiation with 0.05 eq of DBU for 5 min (Table 3, entry 1). This provided the ester **11** in excellent yield as an inseparable mixture of isomers with no diastereomeric excess (50:50).

The addition of nitroethane (**10**) to methyl acrylate (**4**) was carried out for 5 min (Table 3, entry 2). The product **12** was obtained in essentially quantitative yields. Finally, we found that the addition of nitroethane (**10**) to methyl methacrylate (**7**) under microwave irradiation for 5 min (Table 3, entry 3) affords the ester **13**, which was isolated as a 78:22 diastereomeric mixture (by ^1^H-NMR analysis). In this case, the esters were separated by column chromatography without assignment of the absolute configuration. 

**Table 3:** Addition of nitroethane (**10**) to different methyl acrylates (**1**, **4**, and **7**).

Entry	Methyl acrylates	Eq. DBU	Power (W)	Temperature (^o^C)	Time (min)	Product (%) *^a^*
1	**1**	0.05	50	70-75	5	**11** (98) *^b^*
2	**4**	0.05	50	70-75	5	**12** (> 99) *^c^*
3	**7**	0.05	50	70-75	5	**13** (83) *^b^*

*^a^* Yield after their purification by column chromatography. *^b^* Diastereomeric ratio was obtained by ^1^H-NMR. *^c^* When the reaction is carried out for 4,320 min without microwave irradiation the yield was only 44 %.

## 3. Conclusions

In summary, a practical and high-yielding methodology to synthesize γ-nitrobutyric acid methyl esters under microwave irradiation has been developed. With this protocol the reaction times are significantly shortened, in comparison with conventional conditions. The extension of this methodology to other α,β-unsaturated compounds has proven successful and this work is currently under further investigation.

## 4. Experimental

### 4.1. General

^1^H- and ^13^C-NMR spectra were obtained in CDCl_3_ solutions with TMS as internal standard on Varian Gemini 200 or Inova 400 MHz spectrometers. Reactions were performed in sealed vessels in a monomode microwave CEM Discover apparatus and the temperature was evaluated by infrared. Methyl crotonate (**1**), nitromethane (**2**), methyl acrylate (**4**), methyl methacrylate (**7**), nitroethane (**10**), DBU, and TMG were purchased from Aldrich and used without further purification. 

### 4.2. General procedure: Synthesis of methyl 4-nitro alkyl esters via microwave irradiation

A solution of α,β-unsaturated ester (**1**, **4**, **7**) and the corresponding nitroalkane (**2** or **10**) in DBU or TMG were added to a 10-mL glass microwave reaction vessel containing a stir bar. The reaction vessel was sealed with a cap and then placed into the microwave cavity. The microwave unit was programmed to heat the reaction mixture to the desired temperature. After the reaction was completed and the vessel was cooled to below 50 ^o^C using a flow of compressed air, the crude material was purified by column chromatography to give the product.

*Methyl 3-methyl-4-nitrobutanoate* (**3**): Prepared from methyl crotonate (**1**, 10 mmol), nitromethane (**2**, 25 mmol) and DBU (0.1 mmol). The microwave unit was programmed to 70-75 ^o^C and a power of 50 Watts for 5 min. After the reaction was completed, the vessel was cooled to below 50 ^o^C using a flow of compressed air. The crude material was purified by column chromatography (hexane–ethyl acetate 8:2), afforded the product **3** (≥ 98 %) as a colorless liquid. ^1^H-NMR (200 MHz, CDCl_3_) δ 1.11 (d, *J* = 7.0 Hz, 3H), 2.42 (dd, *J* = 6.8, 7.0 Hz, 2H), 2.68-2.90 (m, 1H), 3.70 (s, 3H), 4.35 (dd, *J* = 12.1, 6.9 Hz, 1H), 4.49 (dd, 12.1, 6.2 Hz, 1H); ^13^C-NMR (50 MHz, CDCl_3_) δ 17.6, 29.7, 37.9, 52.1, 80.4, 171.8; HRMS (FAB+): calcd for [M+H]^+^ C_6_H_12_NO_4_: 162.0766; found: 162.0779. 

*Methyl 4-nitrobutanoate* (**5**) *and* *dimethyl 4-nitroheptanedioate* (**6**): According with the general procedure, methyl acrylate (**4**, 10 mmol), nitromethane (**2**, 25 mmol) and DBU (0.05 mmol) were heated at 70-75 ^o^C with a power of 25 Watts for 0.5 h. The crude material was purified by column chromatography (hexane-ethyl acetate 95:5) afforded **5** (69 %) and **6** (10 %) as a colorless liquid. Compound **5**: ^1^H-NMR (200 MHz, CDCl_3_) δ 2.04-2.33 (m, 4H), 2.36-2.49 (m, 2H), 3.69 (s, 3H); ^13^C NMR (50 MHz, CDCl_3_) δ 28.86, 30.10, 52.20, 86.77, 172.32; MS–EI^+^ (m/z, %): 116 (M–OMe). Compound **6**: ^1^H-NMR (200 MHz, CDCl_3_) δ 2.20-2.38 (m, 9H), 3.69 (s, 6H); ^13^C-NMR (50 MHz, CDCl_3_) δ 28.63, 30.40, 52.24, 91.96, 172.20; MS–EI^+^ (m/z, %): 202 (M–OMe).

*Methyl 2-methyl-4-nitrobutanoate* (**8**) *and dimethyl 2,6-dimethyl-4-nitroheptanedioate* (**9**): Prepared from methyl methacrylate (**7**, 20 mmol), nitromethane (**2**, 25 mmol) and TMG (0.5 mmol). The Microwave unit was programmed to 70-75 ^o^C and a power of 25 Watts for 20 min. After the reaction was completed, the vessel was cooled to below 50 ^o^C using a flow of compressed air. The crude material was purified by column chromatography (hexane–ethyl acetate 98:2), afforded the product **8** (72 %) and **9** (28 %) as a colorless liquid. Compound **8**: ^1^H-NMR (200 MHz, CDCl_3_) δ 1.25 (d, *J* = 7.0 Hz, 3H), 2.0-2.43 (m, 2H), 2.50-2.70 (m, 1H), 3.71 (s, 3H), 4.46 (t, *J* = 7.0 Hz, 2H,); ^13^C-NMR (50 MHz, CDCl_3_) δ 17.4, 30.7, 36.6, 52.2, 73.5, 175.3; HRMS (FAB+): calcd for [M+H]^+^ C_6_H_12_NO_4_: 162.0766; found: 162.0863. Compound **9**: ^1^H-NMR (200 MHz, CDCl_3_) δ 1.20 (d, *J* = 5.80 Hz, 3H), 1.24 (d, *J* = 5.80 Hz, 3H), 1.74-1.93 (m, 2H), 2.0-2.11 (m, 2H), 2.35-2.53 (m, 2H), 3.69 (s, 3H), 3.71 (s, 3H), 4.57-4.74 (m, *J* = 4.4 Hz, 1H); ^13^C-NMR (50 MHz, CDCl_3_) δ 17.96, 36.29,37.59, 52.08, 85.41, 175.36; HRMS (FAB+): calcd for [M+H]^+^ C_11_H_20_NO_6_: 262.1246; found: 262.1270.

*Methyl 3-methy-4-nitropentanoate* (**11**): Prepared from methyl crotonate (**1**, 10 mmol), nitroethane (**10**, 25 mmol) and DBU (0.05 mmol). The microwave unit was programmed to 70-75 ^o^C and a power of 50 Watts for 5 min to afford a 1:1 mixture of diastereomeric compounds, which were not separable. The crude material was purified by column chromatography (hexane–ethyl acetate 9:1), afforded the product **11** (98 %) as a colorless liquid. ^1^H-NMR (200 MHz, CDCl_3_) δ 1.03 (dd, *J* = 1.2, 1.6 Hz, 3H), 1.53 (dd, *J* = 3.6, 3.6 Hz, 3H) 2.18–2.30 (m, 1H), 2.35–2.43 (m, 1H), 2.46-2.66 (m, 1H), 3.70 (s, 3H), 4.53–4.70 (m, 1H); ^13^C-NMR (50 MHz, CDCl_3_) δ 15.64, 15.81, 16.55, 34.57, 34.69, 37.30, 37.54, 52.08, 86.57, 86.62, 171.97, 172.17; HRMS (FAB+): calcd for [M+H]^+^C_11_H_20_NO_6_: 176.0878; found: 176.0945.

*Methyl 4-nitropentanoate* (**12**): Prepared from methyl acrylate (**7**, 20 mmol), nitroethane (**10**, 25 mmol) and DBU (0.05 mmol). The microwave unit was programmed to 70-75 ^o^C and a power of 50 Watts for 5 min. After the reaction was completed, the vessel was cooled to below 50 ^o^C using a flow of compressed air. The crude material was purified by column chromatography (hexane–ethyl acetate 80:20), afforded the product **12** (> 99 %) as a colorless liquid. ^1^H-NMR (400 MHz, CDCl_3_) δ 1.57 (d, *J* = 6.8Hz, 3H), 2.06–2.16 (m, 1H), 2.23–2.34 (m, 1H), 2.35-2.47 (m, 2H), 3.70 (s, 3H), 4.62–4.71 (m, 1H); ^13^C-NMR (100 MHz, CDCl_3_) δ 19.2, 29.7, 29.8, 51.8, 82.3, 172.3; HRMS (FAB+): calcd for [M+H]^+^ C_6_H_12_NO_4_: 162.0766; found: 162.0768.

*Methyl 2-methy-4-nitropentanoate* (**13**): Prepared from methyl methacrylate (**7**, 20 mmol), nitroethane (**10**, 25 mmol) and DBU (0.05 mmol). The microwave unit was programmed to 70-75 ^o^C and a power of 50 Watts for 5 min. After the reaction was completed, the vessel was cooled to below 50 ^o^C using a flow of compressed air. The crude material was purified by column chromatography (hexane 100%), afforded a 72:22 diastereomeric mixture of **13** as a colorless liquid. Less polar compound **13**: ^1^H-NMR (200 MHz, CDCl_3_) δ 1.21 (d, *J* = 7.4 Hz, 3H), 1.55 (d, *J* = 6.6 Hz, 3H) 2.02–2.09 (m, 2H), 2.39–2.57 (m, 1H), 3.71 (s, 3H), 4.56–4.73 (m, *J* = 6.8 Hz, 1H); ^13^C-NMR (50 MHz, CDCl_3_) δ 18.17, 20.17, 36.48, 38.70, 52.13, 82.10, 175.74. More polar compound **13**: ^1^H-NMR (200 MHz, CDCl_3_) δ 1.24 (d, *J* = 7.0 Hz, 3H), 1.56 (d, *J* = 6.8 Hz, 3H) 1.75–1.88 (m, 1H), 2.38–2.55 (m, 2H), 3.70 (s, 3H), 4.56–4.75 (m, *J* = 6.6 Hz, 1H); ^13^C-NMR (50 MHz, CDCl_3_) δ 17.10, 19.72, 36.44, 38.38, 52.18, 81.27, 175.47; MS–EI^+^ (m/z, %): 144 (M–OMe).
